# Explainable Deep Learning for MRI-Negative Temporal Lobe Epilepsy: Classification and Brain Region Analysis

**DOI:** 10.3390/diagnostics16172753

**Published:** 2026-08-27

**Authors:** He Wang, Yilin Jiang, Kaiyue Wu, Jiechuan Ren, Wenhan Hu, Zhimei Li, Chunlan Yang, Ying Duan

**Affiliations:** 1 College of Chemistry and Life Science, Beijing University of Technology, Beijing 100124, China; wanghe@emails.bjut.edu.cn (H.W.); jiangyilin1709@163.com (Y.J.); 15522582581@163.com (K.W.); 2 Beijing Tiantan Hospital, Capital Medical University, Beijing 100050, China; jiechuanren@126.com (J.R.); lizm1211@163.com (Z.L.); 3 Department of Neurosurgery, Tiantan Hospital, Beijing 100070, China; huwenhan88@163.com; 4 Beijing Universal Medical Imaging Diagnostic Center, Beijing 100039, China

**Keywords:** temporal lobe epilepsy, MRI-negative, hierarchical, multiscale residual network, layer-wise relevance propagation, explainable artificial intelligence

## Abstract

**Background/Objectives**: Deep learning has achieved remarkable success in medical image analysis; however, limited model interpretability remains a major barrier to its clinical translation. MRI-negative temporal lobe epilepsy (TLE) is characterized by the absence of readily identifiable structural abnormalities on conventional MRI. **Methods:** We propose a hierarchical, multiscale 3D residual network (H-MSResNet) combined with layer-wise relevance propagation (LRP). The study included structural T1-weighted MRIs from 101 patients with MRI-negative TLE and 101 healthy controls. Model classification performance was evaluated using a fivefold cross-validation approach. Subsequently, group-level LRP analysis was integrated with a standard brain atlas to quantify the anatomical regions contributing to the model’s decisions. **Results**: H-MSResNet achieved an average classification accuracy of 76.27% and a best single-fold accuracy of 82.50%, with higher accuracy, specificity, and F1 score but lower sensitivity and AUC than the two comparison models. Group-level, LRP-based analysis combined with a standard brain atlas revealed that the model’s decision-making primarily focused on structures related to the temporal lobe and limbic system, including regions such as the hippocampus, parahippocampal gyrus, and amygdala. Population-level attribution also showed interhemispheric differences across several regions. **Conclusions**: Structural MRIs of MRI-negative TLE contain latent discriminative information that can be recognized by deep learning models. The H-MSResNet and LRP framework provides viable, explainable methodological support for the computer-aided diagnosis and brain region analysis of MRI-negative epilepsy.

## 1. Introduction

Epilepsy is one of the most common neurological disorders worldwide, and the risk of premature death in patients with epilepsy is up to three times higher than that in the general population [1,2]. Temporal lobe epilepsy (TLE) is the most frequent type of focal epilepsy. High-resolution structural magnetic resonance imaging (MRI) plays a critical role in identifying associated structural lesions, such as hippocampal sclerosis and focal cortical dysplasia [3,4].

Approximately 30% of patients with TLE do not present with definitive structural lesions on conventional MRI, a condition commonly referred to as MRI-negative TLE [5,6]. Owing to the lack of visible neuroimaging evidence, clinical evaluation for these patients is highly challenging, and their seizure-free rates following surgical intervention are significantly lower than those of MRI-positive patients [7]. The absence of distinct lesions on conventional MRI does not preclude the existence of subtle structural differences that are imperceptible to the naked eye. Therefore, identifying these potential differences and clarifying their anatomical distribution remains a crucial issue in neuroimaging research on MRI-negative TLE.

To identify these potential structural differences, previous studies utilized various quantitative methods, such as voxel-based morphometry, cortical morphology analysis, and region-of-interest (ROI) morphometry, to investigate the structural MRI of patients with TLE [8,9]. Relevant findings suggest that even when conventional MRI does not reveal definite lesions, patients with MRI-negative TLE may still exhibit subtle alterations in regional morphology, gray matter structure, or gray–white matter boundary within the hippocampus, parahippocampal gyrus, amygdala, and divisions of the mesial and lateral temporal lobes [8,9,10,11]. These findings provide imaging evidence for recognizing potential abnormal brain regions in MRI-negative TLE and suggest that the potential structural changes have a certain degree of individual heterogeneity [12]. However, existing quantitative methods typically rely on predefined morphometric indices or ROIs, which may be insufficient to describe the complex imaging features present in raw 3D MRI. Therefore, methodologies that automatically extract disease-related imaging features from raw 3D MRI and further analyze their distribution across brain regions must be explored.

Deep learning has been widely used in structural MRI-assisted diagnosis because of its end-to-end representation learning capabilities and has shown good performance in classifying various neurological diseases [13,14,15]. Multicenter studies confirmed that 3D convolutional neural networks (3D CNNs) can distinguish patients with MRI-negative TLE from healthy controls using structural MRIs that show no definitive lesions upon routine visual inspection. This finding indicates that such images still contain diagnostically valuable features recognizable by the model [16,17]. However, owing to the lack of explicit lesion annotations in MRI-negative TLE, current models are mostly trained on the categorical labels of patients and healthy controls. Although disease classification can be achieved, the anatomical regions on which the model relies for discrimination are difficult to elucidate. This lack of spatial explainability makes it difficult for classification models to be directly used for the exploration of potential abnormal brain regions [18,19,20]. Therefore, in the absence of explicit lesion annotations, the identification of anatomical regions on which the classification model relies for its judgment is the key to using deep learning to explore potential abnormal brain regions in MRI-negative TLE.

Post hoc explainability methods have been increasingly used in medical imaging to characterize the image information contributing to model predictions. In TLE, saliency-based analysis has been used to visualize the spatial distribution of image features associated with deep-learning classification [17]. Recent peer-reviewed studies have highlighted the diversity of explainable artificial intelligence (XAI) approaches in medical imaging, including saliency- and heatmap-based attribution methods as well as non-saliency-based explanation strategies [21,22]. Importantly, the clinical usefulness of an explanation depends not only on its visual plausibility but also on whether it faithfully reflects the model’s decision process and is relevant to the intended task [23]. However, traditional saliency maps may have high spatial noise [24]. Layer-wise relevance propagation (LRP) distributes model decision scores to the input space through a layer-by-layer backpropagation mechanism, assigning each voxel its contribution to the current prediction results and thereby generating voxel-level relevance maps [25,26]. Previous studies used LRP for the structural MRI classification of neurological diseases such as Alzheimer’s disease to present the spatial distribution of model discrimination criteria in the brain [27,28]. LRP has also been used for hippocampal sclerosis classification to show the brain structural regions that the model focuses on when making judgments [29]. On this basis, combining LRP correlation maps with standard brain atlases can further quantify the contribution of different anatomical regions to model classification decisions, providing a basis for screening disease-related candidate brain regions for MRI-negative TLE.

The attribution results presented by LRP are based on the features learned by the basic classification model. Therefore, brain region attribution analysis first requires the model to effectively extract imaging features with disease-discriminating value from structural MRI. Previous studies suggested that the potential structural differences in MRI-negative TLE are relatively subtle [9,10], and the structural changes in different patients can present local or widespread features [12]. Compared with single-scale convolution, multiscale convolution can learn the complementary spatial information of focal and diffuse pathological features by establishing feature extraction branches with different receptive fields and has been applied in medical image analysis [30,31]. In epilepsy research, multiscale deep learning was applied to a multimodal MRI analysis of drug-resistant epilepsy in children to assist in localizing the regions associated with the epileptogenic zone [32]. By fully extracting disease-discriminating information, the combination of multiscale feature extraction with LRP attribution analysis is expected to aid in further analyzing the distribution of brain regions the model depends on for its discrimination.

Accordingly, this study developed an explainable analysis framework for MRI-negative TLE that integrates hierarchical, multiscale 3D feature learning with LRP-based attribution analysis. Voxel-level relevance maps were aggregated at the population level and mapped to a standard brain atlas to quantify the anatomical distribution of information contributing to model classification. Through this framework, we aimed to characterize model-relevant regional patterns in MRI-negative TLE and to identify candidate brain regions for further neuroimaging investigation.

## 2. Materials and Methods

**Figure 1:**
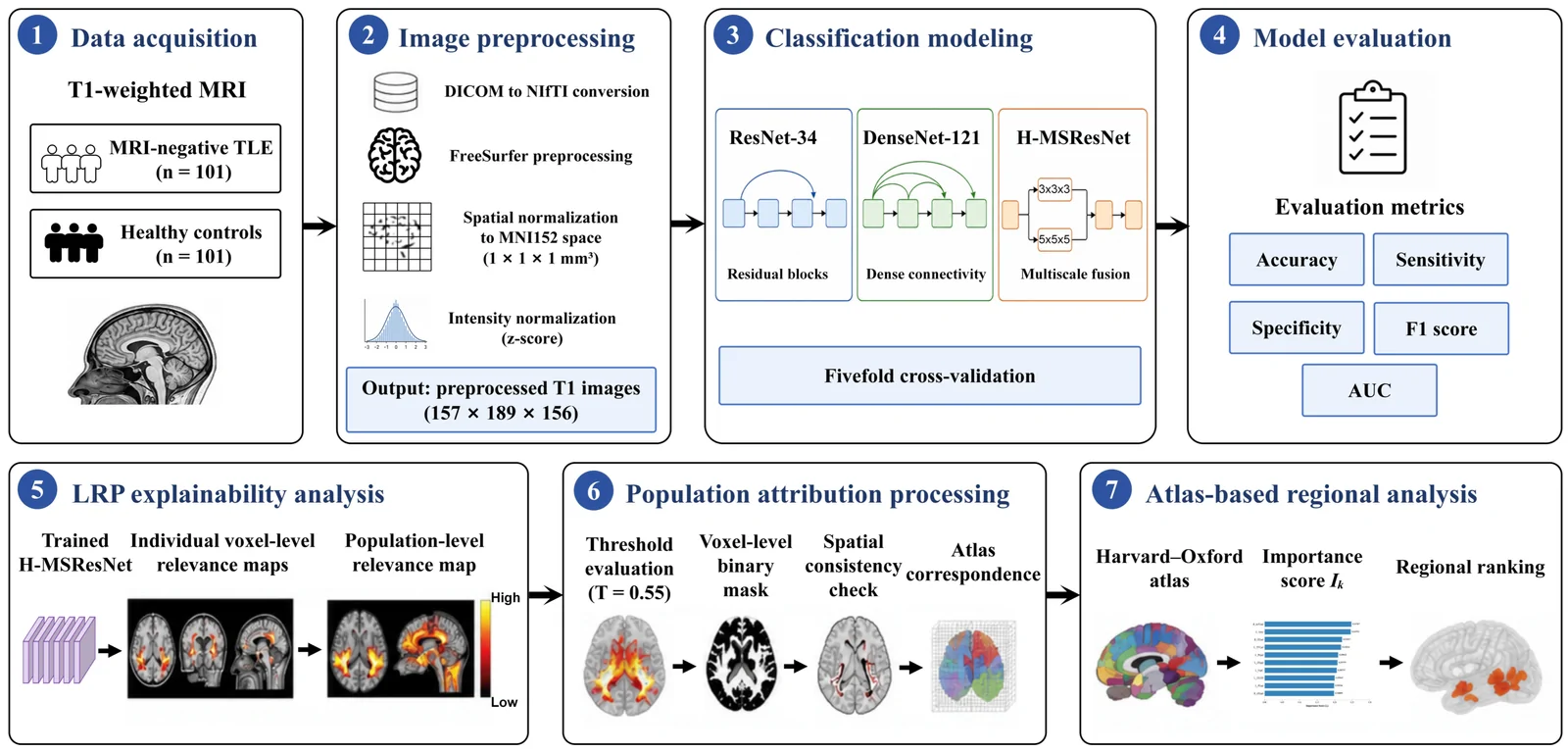
Overall methodological workflow of the proposed explainable deep learning framework for MRI-negative temporal lobe epilepsy. Arrows indicate the sequential flow of the analytical procedure, while colors are used solely for visual differentiation and do not convey additional information.

The overall methodological workflow of this study is illustrated in Figure 1. Briefly, structural T1-weighted MRI data were first preprocessed and subsequently used for classification using three 3D CNN models with fivefold cross-validation. H-MSResNet was further analyzed using layer-wise relevance propagation (LRP) to generate voxel-level relevance maps. Individual relevance maps were then aggregated at the population level and combined with the Harvard–Oxford Atlas for regional importance quantification and spatial distribution analysis.

### 2.1. Data Acquisition

Data were obtained from the Beijing Universal Medical Imaging Diagnostic Center. A total of 202 participants were included in this study, comprising 101 patients with MRI-negative TLE and 101 healthy controls. TLE was clinically diagnosed based on comprehensive clinical assessment and electroencephalographic findings. All patients underwent clinical MRI examinations on a 3.0-T MAGNETOM Prisma scanner (Siemens Healthineers, Erlangen, Germany), including T1-weighted, T2-weighted, FLAIR, and 3D-SPACE sequences. MRI-negative status was defined as the absence of definite structural abnormalities involving the temporal lobes or hippocampi, including hippocampal sclerosis, abnormal signal intensity, atrophy, focal cortical dysplasia, or mass lesions, based on independent assessment by two experienced neuroradiologists. These multiple structural MRI sequences were used for the clinical assessment of MRI-negative status, whereas only the T1-weighted images were used as input for the computational analysis in the present study. The demographic and available clinical characteristics of the study population are summarized in Table 1. No significant between-group differences were observed in age or sex (all *p* > 0.05). All participants signed written informed consent forms, and the study protocol was approved by the Beijing University of Technology Ethics Committee.

T1-weighted images were obtained using a fast gradient echo sequence prepared by magnetization under the following parameters: repetition time = 2300 ms, echo time = 2.3 ms, inversion time = 900 ms, flip angle = 8°, field of view = 256 × 256 mm^2^, resolution matrix = 256 × 256, slices = 192, and slice thickness = 0.9 mm.

### 2.2. Data Preprocessing

**Figure 2:**
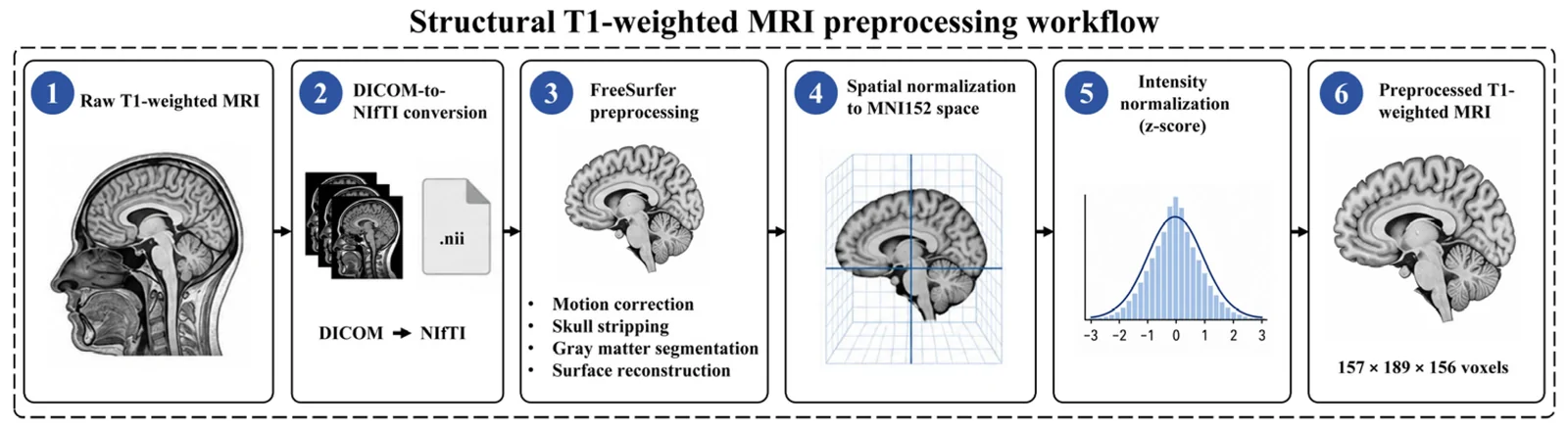
Workflow of structural T1-weighted MRI preprocessing. Original DICOM images were converted to NIfTI format, processed using FreeSurfer, spatially normalized to MNI152 space using SPM12, and subsequently subjected to global intensity normalization. The resulting preprocessed T1-weighted images had dimensions of 157 × 189 × 156 voxels.

The original T1-weighted images in DICOM format were first converted into NIfTI format using SimpleITK (version 2.5.2). The converted data were preprocessed using the recon-all command of FreeSurfer software (version 7.1.1; http://surfer.nmr.mgh.harvard.edu/fswiki/DownloadAndInstall/; accessed on 25 March 2026) [33,34,35]. This procedure included motion correction, nonbrain tissue resection, coordinate transformation, gray matter segmentation, and gray or white matter boundary reconstruction. The resulting skull-stripped image size was 256 × 256 × 256. The T1-weighted images were spatially normalized using the SPM12 (revision 7771) in MATLAB R2017b (MathWorks, Natick, MA, USA; https://www.fil.ion.ucl.ac.uk/spm/; accessed on 25 March 2026) to compare brain images from different individuals. Individual images were nonlinearly registered to the MNI152 standard space to obtain normalized brain images with a size of 157 × 189 × 156 voxels, thus eliminating the interference of interindividual anatomical differences on subsequent deep learning model training. After spatial standardization, global intensity normalization was performed on all images to adjust the voxel intensity distribution to zero mean and unit variance, thereby eliminating the interference of signal intensity differences between individuals on model training. The structural T1-weighted MRI preprocessing procedure is summarized in Figure 2.

### 2.3. Network Architecture and Training Strategy

This study constructed and compared the performance of 3D CNNs in the MRI-negative temporal lobe epilepsy classification task: 3D Residual Network (ResNet-34) [36,37], 3D Densely Connected Convolutional Network (DenseNet-121) [38], and the hierarchical, multiscale 3D ResNet (H-MSResNet) used in this work.

**Figure 3:**
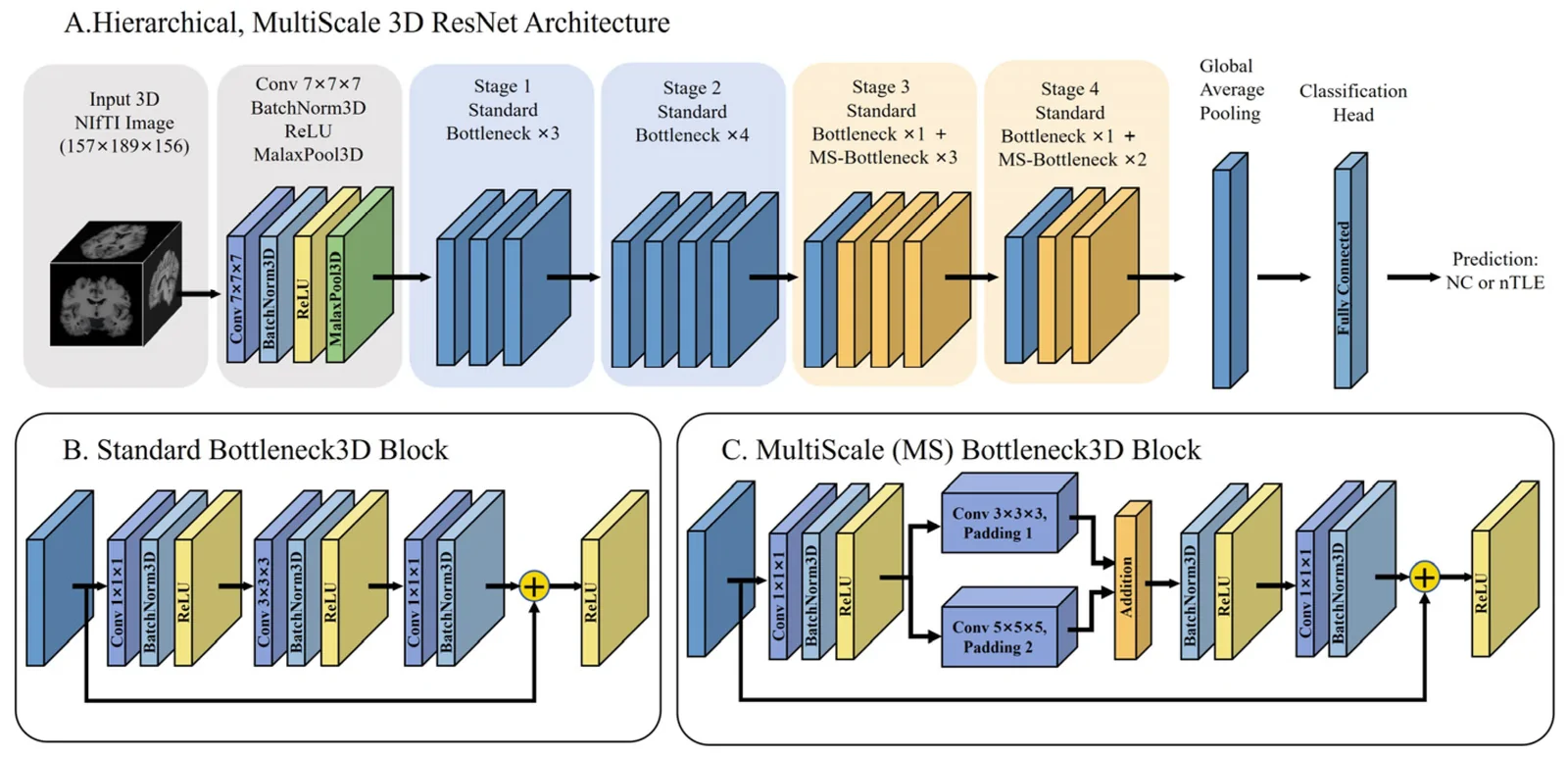
Hierarchical, multiscale 3D ResNet architecture. (**A**) The complete model network architecture, including the input, initial convolutional and pooling layers, four main ResNet stages, global average pooling layers, and the final classification head. (**B**) (Standard bottleneck 3D block) details the standard residual block structure used in the first two layers of the model. (**C**) (Multiscale (MS) bottleneck 3D block) details how the two parallel convolutions of 3 × 3 × 3 and 5 × 5 × 5 perform channel addition fusion. Arrows indicate forward information flow through the network.

H-MSResNet employs a hierarchical, multiscale architecture to adapt to the pathological spatial heterogeneity of MRI-negative TLE. The network structure is shown in Figure 3. Its core design is a hierarchical strategy combining shallow standard convolutions and deep multiscale convolutions. The shallow layers (layers 1–2) use standard 3 × 3 × 3 convolutions to extract focal features, and the deep layers (layers 3–4) introduce parallel, multiscale convolution branches. Here, 3 × 3 × 3 and 5 × 5 × 5 receptive field convolution kernels are used, and multiscale information fusion is achieved through feature addition, enabling the simultaneous perception of focal and diffuse lesions.

In terms of specific implementation, the network input size is 1 × 157 × 189 × 156. After an initial 7 × 7 × 7 convolution and max pooling, the data sequentially pass through four residual stages. The first two stages consist of stacked, standard bottleneck modules. The latter two stages employ a hybrid structure: the first bottleneck module in each stage handles downsampling, and subsequent modules are replaced with multiscale bottleneck modules. The multiscale bottleneck uses 3 × 3 × 3 convolutions connected in parallel with 5 × 5 × 5 convolution branches, summing the two outputs before inputting them into the next layer. The network ultimately outputs binary classification probabilities through global average pooling and fully connected layers.

For fair comparisons across different architectures, all three models employed the same training strategy: fivefold cross-validation. Each run included one fold as validation samples and the remaining four folds as training samples. The optimizer was Adam (initial learning rate 1 × 10^−4^, weight decay 5 × 10^−4^), which employed cosine annealing for learning rate decay. The loss function was the cross-entropy function, the batch size was 8, the training lasted 60 epochs, and the early stopping patience value was 15. All deep learning models were implemented in Python 3.9.21 using PyTorch 2.5.1. To further analyze the distribution of brain regions the model depends on for its classification decisions, this study introduced interpretability analysis methods based on the classification model.

### 2.4. LRP Explainability Analysis

To reveal the anatomical structures upon which the classification model relies in its decision-making, this study employed LRP for attribution analysis on a trained 3D CNN, identifying the key brain regions the model depends on for its decisions. On the basis of the principle of correlation conservation, LRP propagated the model’s output scores back to the input space layer by layer, assigning each voxel its contribution value to the classification decision [25,26,39].

In the LRP implementation, the ε rule (ε = 1 × 10^−8^) was used for convolutional layers to ensure numerical stability:

(1) $$R_{j}^{\left(l\right)}=\sum_{k}\frac{a_{j}^{\left(l\right)}w_{jk}^{\left(l\right)}}{\sum_{j}a_{j}^{\left(l\right)}w_{jk}^{\left(l\right)}+\epsilon }R_{k}^{\left(l+1\right)}$$

where $R_{j}^{\left(l\right)}$ is the relevance score of the j-th neuron in the l-th layer, $a_{j}^{\left(l\right)}$ is the activation value of the neuron during forward propagation of the model, $w_{jk}^{\left(l\right)}$ is the weight connecting the j-th neuron in the l-th layer and the k-th neuron in the (l + 1)-th layer, $\sum_{j^{\prime }}a_{j^{\prime }}^{\left(l\right)}w_{j^{\prime }k}^{\left(l\right)}$ represents the total weighted input received by the k-th neuron in the (l + 1)-th layer from all neurons in the previous layer, ϵ is the numerical stability term (set to 1 × 10^−8^ in this study), used to prevent the denominator from being zero and improve numerical stability during relevance propagation, and $R_{k}^{\left(l+1\right)}$ is the relevance score of the k-th neuron in the (l + 1)-th layer. The same LRP rule and stabilization parameter were applied consistently across all subjects and cross-validation folds.

For residual connections, only positive correlations were retained for propagation. Other layer types (such as pooling layers, fully connected layers, and batch normalization layers) were adapted to the corresponding LRP rules according to their forward propagation characteristics.

After fivefold cross-validation was completed, individual LRP heatmaps were generated for all trained patients with TLE. The relevance value of voxel v in the individual heatmap of the i-th patient is denoted by $R_{i}(v)$, and a positive value indicates that the voxel has a positive contribution to the model’s judgment of the “patient” category.

A population-level brain region importance map was constructed using a weighted relevance aggregation strategy. First, the number of patients’ heatmaps for which each voxel has a positive relevance value (i.e., the relevance value is greater than 0) was counted and recorded as the frequency $N_{pos}(v)$. Second, the cumulative positive relevance value $\sum_{i=1}^{N}R_{i}(v)$ across all patients’ heatmaps was calculated. Finally, the frequency was multiplied by the cumulative sum [40]

(2) $$R_{group}(v)=N_{pos}(v)\times \sum_{i=1}^{N}R_{i}(v)$$

While preserving the intensity of voxel contributions, this strategy assigns high weights to frequently occurring voxels. The population relevance map was obtained and then normalized to the range of [0, 1] for subsequent thresholding.

### 2.5. Brain Region Localization and Quantitative Analysis

#### 2.5.1. Anatomical Atlas Resampling and Spatial Correspondence

Standard brain atlases were further combined for analysis to transform the voxel-level attribution results into anatomical regional distributions. The Harvard–Oxford Cortical and Subcortical Structural Atlas included in the FMRIB Software Library (FSL; version 6.0.7.14) was used as an anatomical template. This atlas contains 48 cortical brain regions (48 in each hemisphere) and 21 subcortical brain regions. After the ventricles and white matter regions were removed, 111 structural brain regions were included for the analysis. The original atlas was located in the MNI152 standard space with a resolution of 182 × 218 × 182. The nearest-neighbor interpolation method in the Nilearn (version 0.12.1) was used for resampling [41] to preserve the discrete brain region labels and match the atlas to the target image dimensions of 157 × 189 × 156. For possible slight coordinate system deviations, the coordinate offset was adjusted by calculating the brain tissue center. After resampling and fine-tuning, rigorous quality verification was performed: (1) the brain tissue overlap rate reached 99.82%, indicating that the brain tissue regions of the atlas and individual images were highly consistent; (2) the number of voxels in key brain regions (such as hippocampus, amygdala, and temporal pole) was consistent with the original atlas (100% retention rate), confirming that the resampling completely preserved the core brain regions related to epilepsy research; and (3) all 111 brain regions were preserved without information loss. The above verification results show that the resampled atlas and individual images achieved good voxel-level correspondence in the MNI space.

#### 2.5.2. Threshold-Based High-Relevance Voxel Extraction

On the basis of the obtained group-weighted heatmaps $R_{group}$, a global thresholding method was used to generate a voxel-level binary mask M(v) to extract the core regions that significantly contribute to the model’s decision-making from these heatmaps and eliminate the interference of background noise. The processing flow was as follows:

First, an empty mask matrix identical in size to the group average heatmap (157 × 189 × 156 dimensions) was created. Threshold evaluation was performed for each voxel: if the normalized heatmap value of the voxel was higher than a preset threshold, then a value of 1 was assigned to its corresponding position in the mask (marking it as an important voxel); otherwise, it was left at 0. This process was formally represented as

(3) $$M(v)=\{\begin{array}{ll} 1, & if\;R_{group}(v)>T\\ 0, & otherwise \end{array}$$

where $R_{group}(v)$ is the normalized value of voxel *v* in the population average heatmap, and T is the screening threshold.

The selection threshold *T* requires consideration because different thresholds retain different amounts of positive relevance information. A higher threshold retains fewer voxels, whereas a lower threshold includes a larger number of voxels. Because the resulting regional attribution may depend on the choice of *T*, thresholds ranging from 0.10 to 0.90 in increments of 0.05 were examined. The threshold dependence of the regional attribution results was subsequently evaluated based on the regional importance scores described in Section 2.5.3. The resulting voxel-level binary masks were used for quantitative analysis of brain region importance, with suprathreshold voxels representing locations with relatively high positive relevance to the model decision.

#### 2.5.3. Brain Region Importance Quantification

The generated binary masks were combined with the registered Harvard–Oxford Atlas. The proportion of voxels with a value of 1 in the binary mask within each brain region out of the total number of voxels in that brain region was calculated. This proportion was used as the brain region importance score, and the regions were then sorted in descending order.

For the *k*-th brain region, its importance score $I_{k}$ was defined as the proportion of important voxels within that brain region out of the total number of voxels in that brain region:

(4) $$I_{k}=\frac{\sum_{v\in R_{k}}M(v)}{\left|R_{k}\right|}$$

where $R_{k}$ is the total number of voxels in brain region *k*, and M(v) is the binary mask. A higher $I_{k}$ indicates that a larger proportion of voxels within the corresponding region exhibit suprathreshold positive relevance to the model decision. All 111 brain regions were sorted in descending order according to their $I_{k}$ values to obtain a ranking of brain region importance.

To formally evaluate threshold sensitivity, the distribution of $I_{k}$ across all 111 brain regions was summarized using the mean, median, and interquartile range (IQR) at each threshold. In addition to the coarse analysis from *T* = 0.10 to *T* = 0.90, a fine-grained analysis was performed from *T* = 0.500 to *T* = 0.600 in increments of 0.005. The consistency of regional rankings between adjacent thresholds was quantified using Spearman rank correlation and Top-10 and Top-20 overlap.

## 3. Results

### 3.1. Classification Model Performance

This study compared the performance of three 3D CNNs on a classification task for MRI-negative TLE. The results of the fivefold cross-validation are shown in Table 2.

In terms of classification accuracy, the average accuracy (76.27%) and best accuracy (82.50%) of H-MSResNet were higher than those of ResNet-34 (74.26%, 75.61%) and DenseNet-121 (74.74%, 80.00%). H-MSResNet also achieved the highest specificity (0.7919) and F1 score (0.7531) among the three models. However, its sensitivity (0.7333) was lower than that of ResNet-34 (0.7705) and DenseNet-121 (0.7533), and its AUC (0.7415) was also lower than that of ResNet-34 (0.7766) and DenseNet-121 (0.7820). These results indicate that H-MSResNet showed advantages in accuracy, specificity, and F1 score, but not across all evaluation metrics.

### 3.2. Population-Level LRP Brain Region Importance Analysis

**Figure 4:**
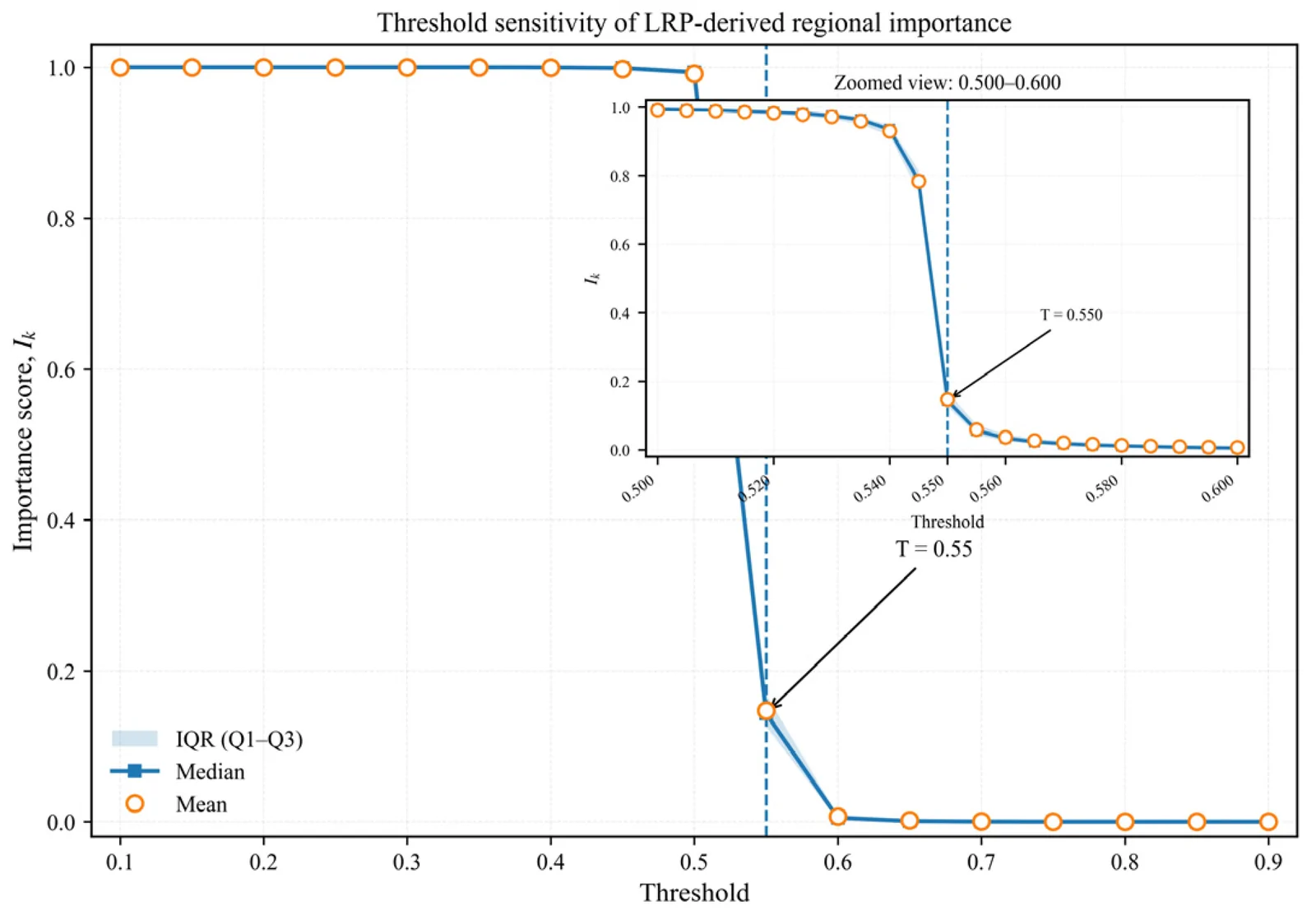
Threshold sensitivity of LRP-derived regional importance scores. Regional importance scores ($I_{k}$) were evaluated across T = 0.10–0.90. The solid line with square markers represents the median $I_{k}$ across 111 brain regions, open circles indicate the mean $I_{k}$, and the shaded area denotes the interquartile range (IQR; 25th–75th percentiles). The inset shows the fine-grained analysis from T = 0.500 to T = 0.600 in increments of 0.005. The dashed vertical line marks T = 0.55.

We generated individual-level LRP correlation heatmaps for MRI-negative TLE patients after training. Population-level heatmaps were obtained using a weighted relevance aggregation strategy. To evaluate the sensitivity of regional attribution to threshold selection, regional importance scores ($I_{k}$) were examined across the threshold range of *T* = 0.10–0.90. The coarse analysis revealed a pronounced threshold-dependent change in the distribution of $I_{k}$. At lower thresholds, the regional scores showed a marked ceiling effect. For example, at *T* = 0.50, all 111 brain regions had $I_{k}$ ≥ 0.95. Fine-grained analysis from *T* = 0.500 to *T* = 0.600 further localized the largest distributional change to the interval between *T* = 0.545 and *T* = 0.550. The mean $I_{k}$ decreased from 0.7835 to 0.1473, while the median decreased from 0.7855 to 0.1437. The corresponding IQR shifted from 0.7529–0.8164 to 0.1265–0.1690, indicating a broad change in the regional-score distribution, as shown in Figure 4.

**Figure 5:**
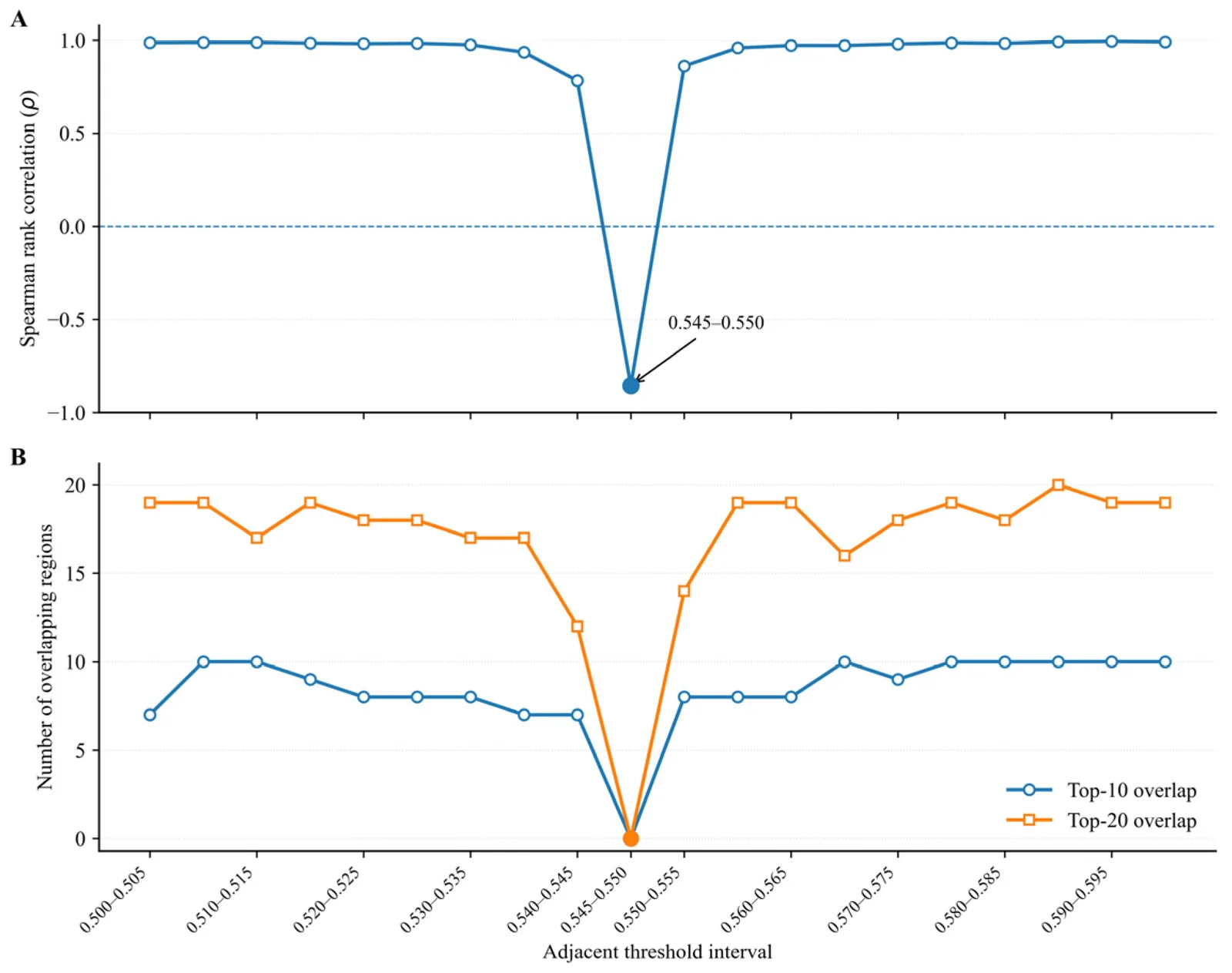
Stability of regional rankings across adjacent thresholds. (**A**) Spearman rank correlations between regional importance-score ($I_{k}$) vectors at adjacent thresholds. (**B**) Number of overlapping regions among the Top-10 and Top-20 rankings between adjacent thresholds.

Consistent with the distributional change, the regional rankings changed substantially between *T* = 0.545 and *T* = 0.550, with a Spearman rank correlation of *ρ* = −0.855. No overlap was observed between the Top-10 or Top-20 regions at these two thresholds. After this interval, the rankings between adjacent thresholds became more consistent. Beginning with the comparison between *T* = 0.555 and *T* = 0.560, Spearman correlations between adjacent thresholds remained above 0.95, accompanied by high Top-10 and Top-20 overlap (Figure 5). These results indicate that regional rankings were sensitive to threshold selection, with the most pronounced change occurring between *T* = 0.545 and *T* = 0.550, followed by greater consistency between adjacent thresholds at higher thresholds.

Regional results reported below were based on *T* = 0.55 as the reference threshold, while the sensitivity analysis above was used to characterize the dependence of regional scores and rankings on threshold selection.

**Figure 6:**
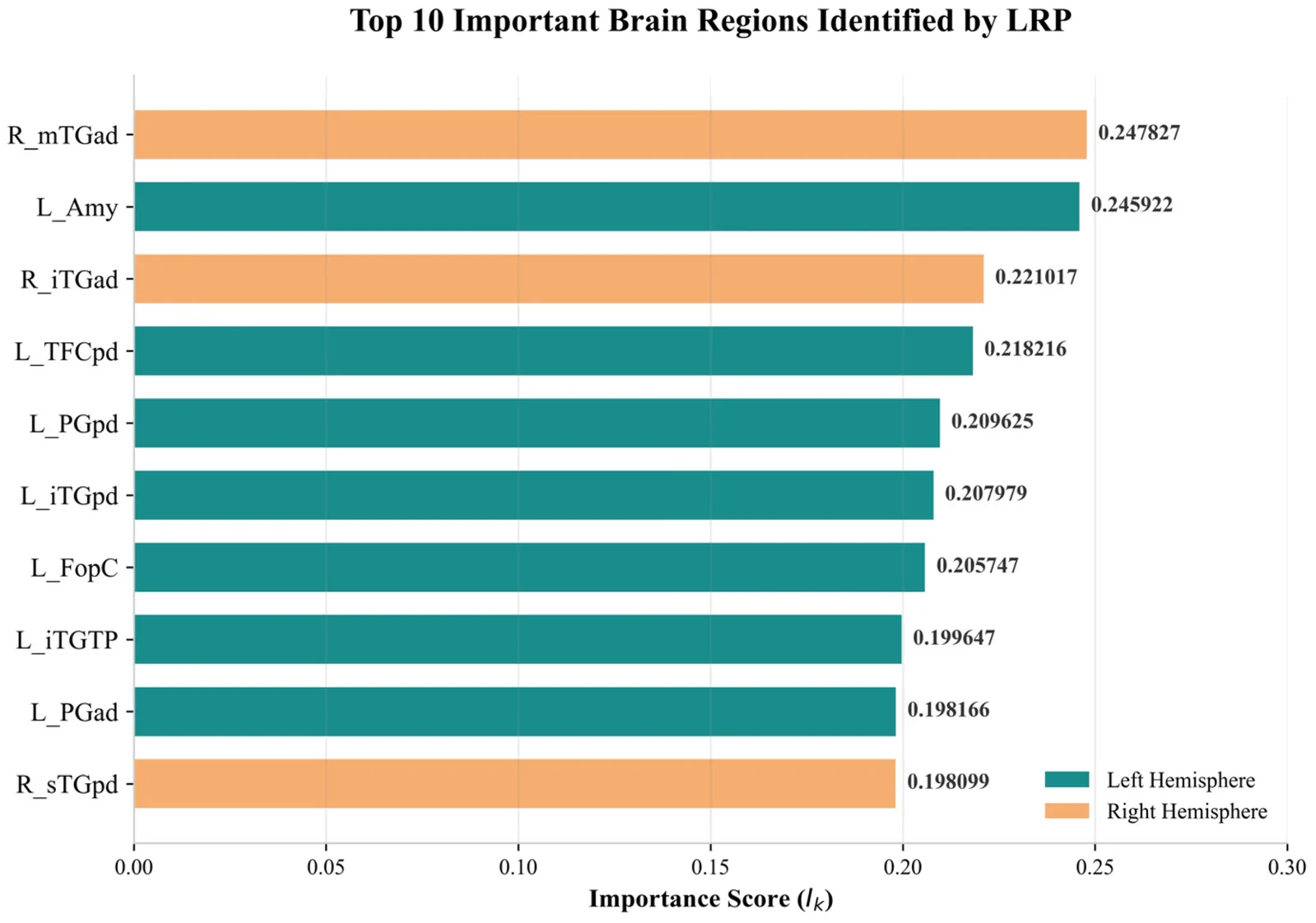
Bar chart of the top ten brain regions ranked by importance scores. The dark cyan bars represent the left hemisphere brain area, and the sandy brown bars represent the right hemisphere brain area.

**Figure 7:**
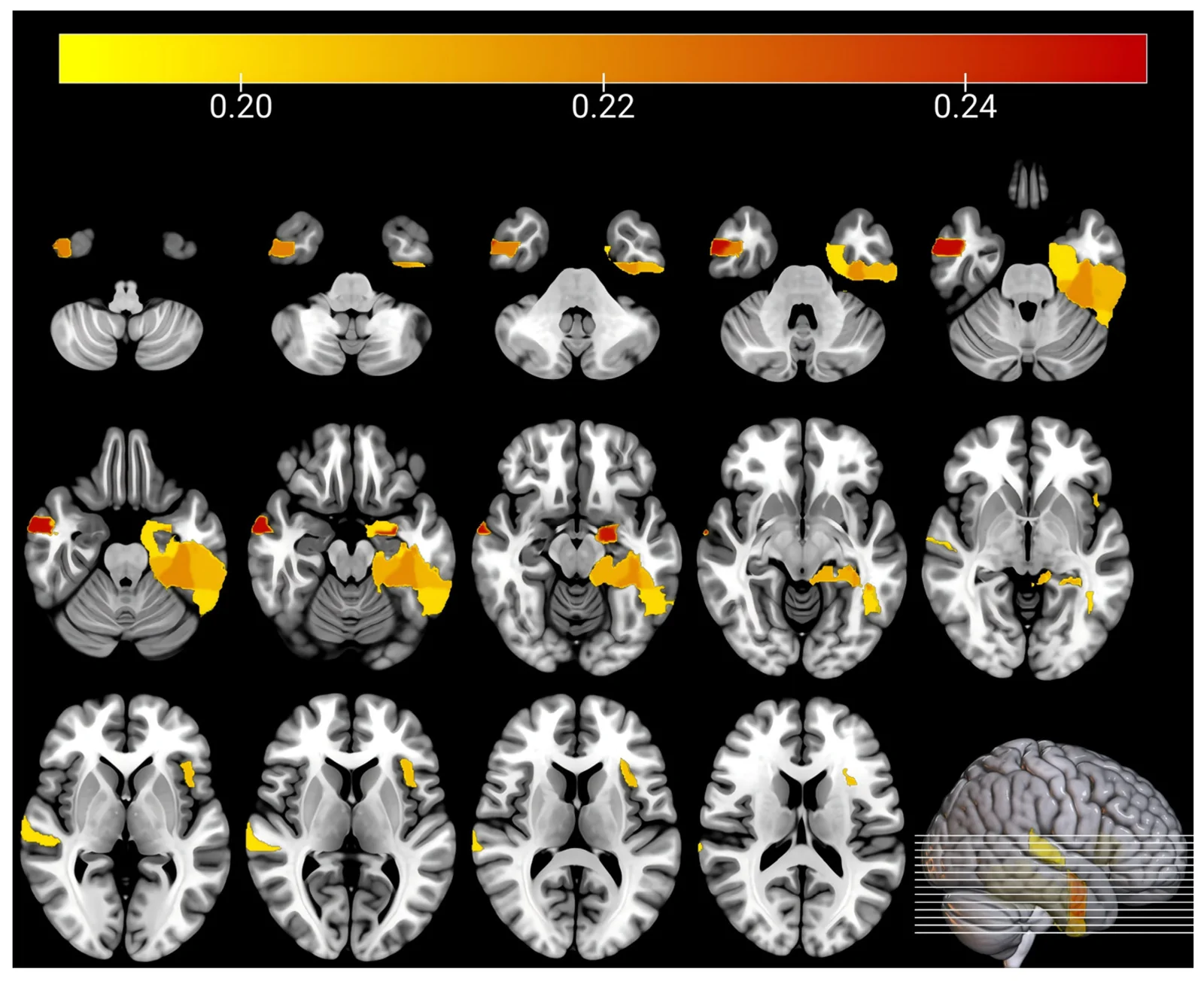
Projections of heatmaps of the top ten brain regions in importance score onto a standard brain template. The color bars represent $I_{k}$ values, with red indicating high importance and yellow indicating low importance. The horizontal lines in the sagittal view are slice-position reference lines indicating the locations of the axial slices displayed in the figure.

Among the top 10 brain regions in importance score, the anterior division of the right middle temporal gyrus (R_mTGad) (0.2478) ranked first, the left amygdala (L_Amy) (0.2459) ranked second, and the anterior division of the right inferior temporal gyrus (R_iTGad) (0.2210) ranked third (Figure 6). Nine of the top ten brain regions were located in the temporal lobe and its medial structures, including the amygdala, parahippocampal gyrus, inferior temporal gyrus, anterior division of the middle temporal gyrus, posterior division of the superior temporal gyrus, and posterior division of the temporal fusiform cortex. One region was located in the left frontal operculum cortex in the frontal lobe. The projection of the heatmap of the top 10 brain regions in importance score onto a standard brain template is shown in Figure 7.

**Figure A1:**
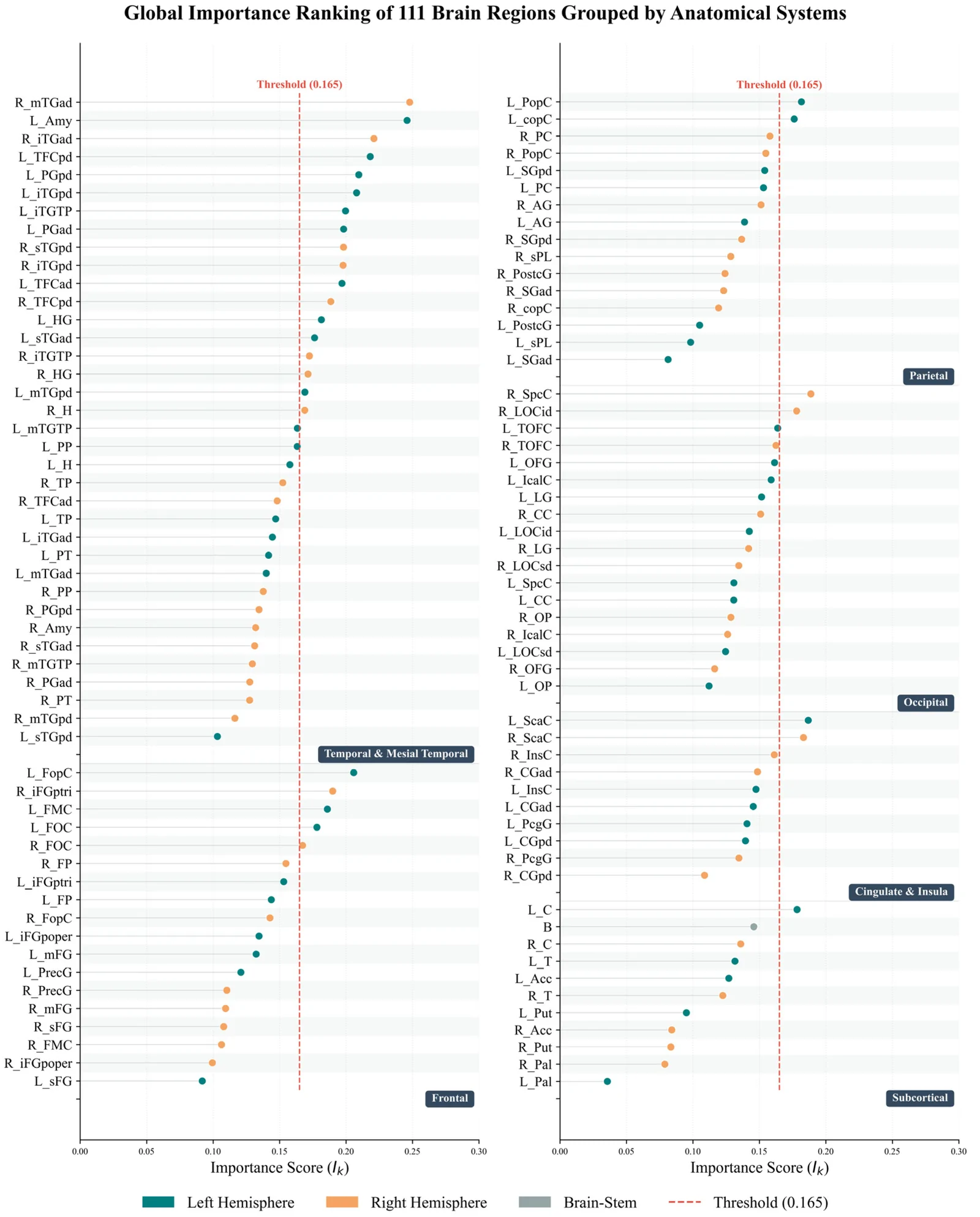
Ranking of the importance score ($I_{k}$) of the whole-brain anatomical regions based on the Harvard–Oxford Atlas. The brain regions are divided into six major systems according to conventional anatomical definitions and displayed in two columns. The left column includes the temporal lobe, the medial temporal lobe system and the frontal lobe system. The right column includes the parietal lobe, occipital lobe, cingulate gyrus and insula, and subcortical structures. In the figure, dark cyan scatter dots represent the left hemisphere brain regions, sandy brown scatter dots represent the right hemisphere brain regions, and gray scatter dots represent the brainstem. The red dashed line represents the importance threshold (threshold = 0.165), and to its right are the top 30 core brain regions that make key contributions to classification decisions.

When viewed as the whole brain, the temporal lobe and medial limbic system dominated in importance scores (Figure A1). Among the top 10 brain regions, 9 were located in the temporal lobe and its medial region. Among the top 20, 15 were located in the temporal lobe and its medial region. Among the top 30, 18 were located in the temporal lobe and its medial region. Among the top 50, 27 were located in the temporal lobe and its medial region. In addition to the core temporal lobe system, the frontal lobe system (such as the left insular cortex and the right inferior frontal gyrus triangle) and some limbic structures (such as the left subcallosal cortex) also exhibited high decision weights, revealing a complex whole-brain involvement pattern in MRI-negative TLE.

**Figure 8:**
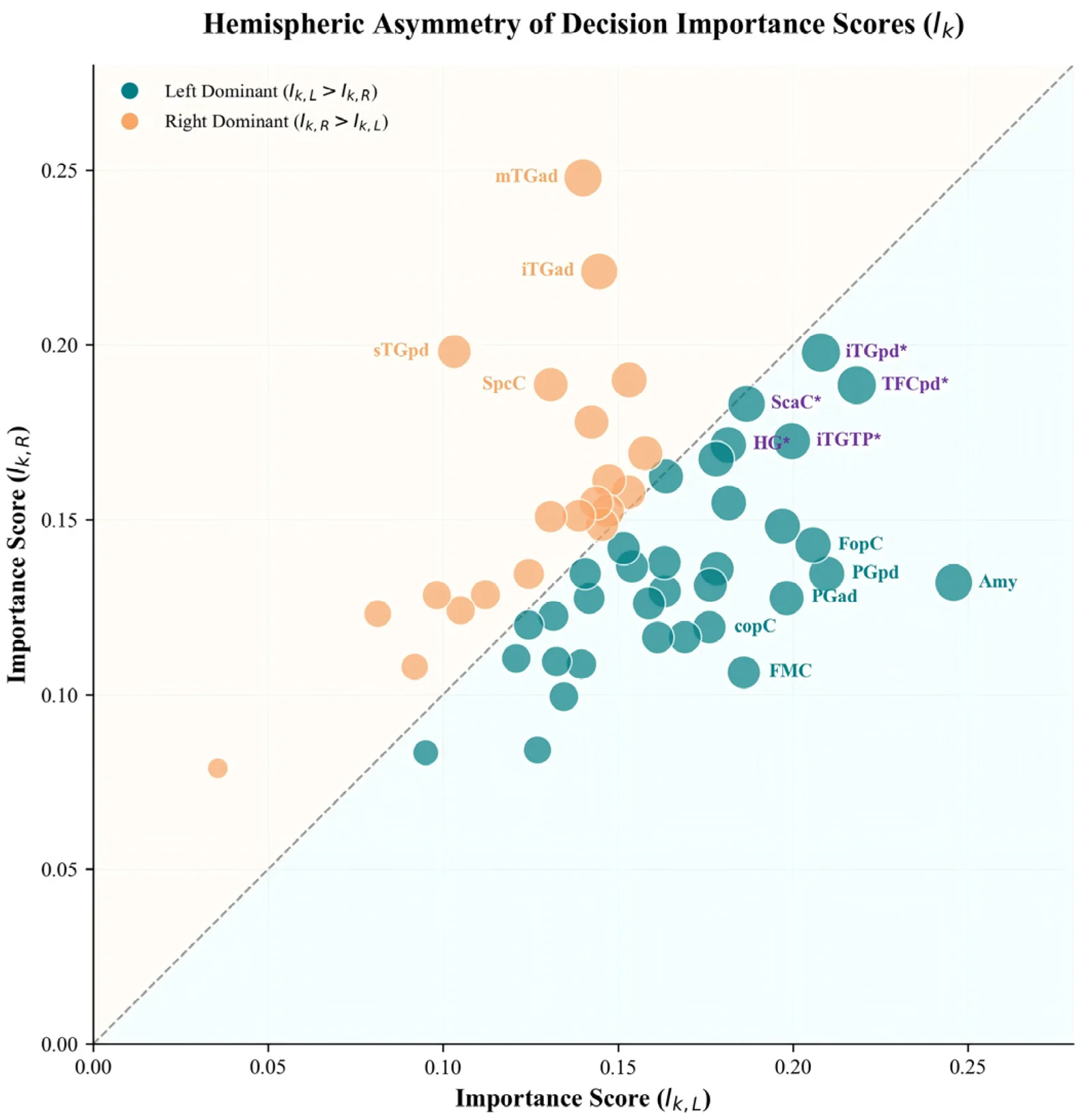
Hemispheric asymmetry and distribution characteristics of decision importance scores ($I_{k}$). The figure shows the correlation distribution of importance scores ($I_{k,L}$ vs. $I_{k,R}$) of 55 paired brain regions in the left and right hemispheres. The asymmetry of decision weights was quantified by the degree of deviation from the diagonal (y=x). Dark cyan and sand ochre regions represent brain regions that are dominant in the left and right hemispheres, respectively. The dark cyan and sand ochre labels mark the top 10 brain regions with the largest lateral differences ($\left|I_{k,L}-I_{k,R}\right|$). Purple region labels followed by an asterisk (*) indicate the top five brain regions with relatively balanced distribution but the highest overall importance scores. The scatter plot size represents the total bilateral importance score.

The comparison of 55 paired brain regions (Figure 8) revealed interhemispheric differences in population-level attribution scores. Relatively higher left-sided attribution scores were observed in several limbic structures, including the amygdala and the anterior and posterior divisions of the parahippocampal gyrus. In contrast, relatively higher right-sided attribution scores were observed in regions such as the anterior division of the middle temporal gyrus. Several regions also showed relatively balanced bilateral contributions. These findings reflect interhemispheric differences in population-level model attribution within the pooled MRI-negative TLE cohort, while their relationship with seizure-onset laterality remains undetermined.

**Figure 9:**
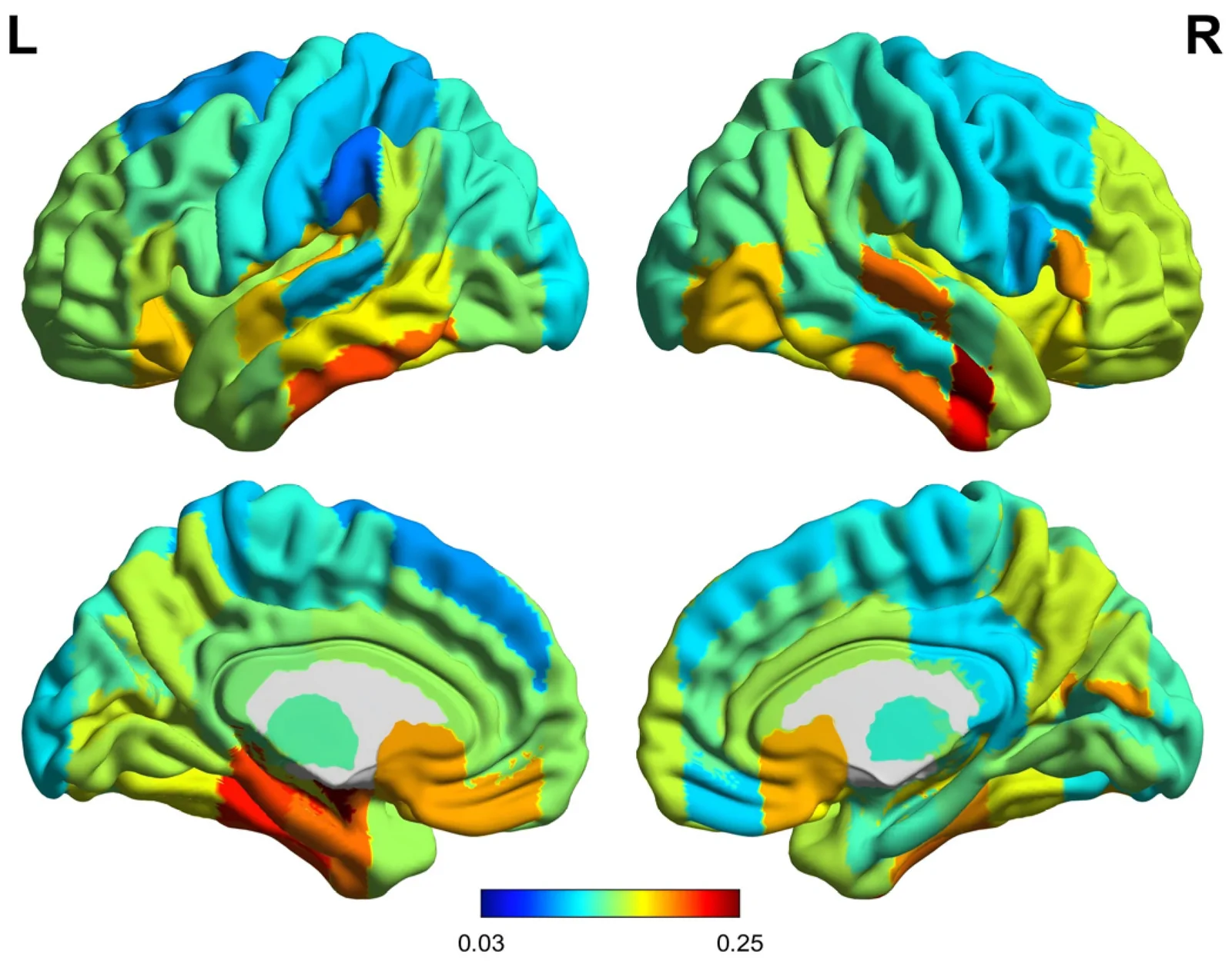
Heatmap of whole-brain cortical surface projection distribution based on population-level correlation weights. In the figure, the colors represent brain region importance scores ($I_{k}$), generated by mapping the statistical scores of all brain regions to a standard cortical template. Red areas represent brain regions with high $I_{k}$, and blue areas represent brain regions with low $I_{k}$.

Finally, to characterize the whole-brain topological distribution of decision weights at a macroscopic level, this study projected population-level brain region importance scores onto a standard cortical surface (Figure 9). The high-scoring regions were mainly concentrated in the bilateral medial temporal lobes, including L_Amy, the bilateral parahippocampal gyrus, and the anterior division of the right middle temporal gyrus. The visualization results were generally consistent with the brain region importance scores (Figure A1). In limbic system structures such as the amygdala and parahippocampal gyrus, the left side showed relatively higher contribution values, while R_mTGad exhibited a higher importance score. These results indicate that the model classification process exhibits certain lateral differences and spatial distribution characteristics in different brain regions.

## 4. Discussion

This study constructed an explainable analytical framework combining a hierarchical, multiscale 3D residual network and LRP for the classification of MRI-negative TLE and the characterization of model-relevant regional patterns.

The findings of this study reveal three key points. First, H-MSResNet achieved higher mean accuracy, specificity, and F1 score than the two comparison models, whereas its sensitivity and AUC were lower. Second, the population LRP attribution results were mainly distributed in the medial and lateral temporal lobes, with the anterior division of the right middle temporal gyrus, L_Amy, and the parahippocampal gyrus showing high classification contributions. Third, the contribution distribution of different candidate brain regions was not entirely consistent between the left and right hemispheres, with unilateral and bilateral regions showing high contributions. These results indicate that structural MRI without definite lesions on routine visual examination still contains disease-discriminating features that can be recognized by deep learning models, and these features exhibit a predominantly temporal lobe-based anatomical distribution in the model’s attribution results.

### 4.1. Multiscale Architecture Captures Subtle Imaging Features of MRI-Negative Temporal Lobe Epilepsy

The H-MSResNet proposed in this study achieved an average accuracy of 76.27% and a best single-fold accuracy of 82.50% in the classification task of MRI-negative temporal lobe epilepsy, with an average accuracy higher than those of ResNet-34 (74.26%) and DenseNet-121 (74.74%). Recent multicenter studies provide relevant benchmarks for MRI-negative TLE classification using structural MRI. Kaestner et al. evaluated a 3D CNN using 1178 T1-weighted MRI scans from 12 centers and reported a median overall accuracy of 86.4%, with an accuracy of 82.7% in the MRI-negative subgroup [16]. In a subsequent analysis focusing on surgically validated patients from this multicenter cohort, Gleichgerrcht et al. also reported an accuracy of 82.7% for MRI-negative TLE [17]. These values were higher than the mean accuracy of H-MSResNet in the present study (76.27%). The higher accuracies reported in these studies should be interpreted in the context of the substantially larger multicenter dataset, as well as differences in cohort definition and validation procedures relative to the present study.

From the perspective of network architecture design, the accuracy improvement of H-MSResNet compared with the two baseline models may be related to its multiscale feature extraction. Previous studies showed that MRI-negative patients with TLE may have subtle regional morphological and gray matter structure differences [9,10], and their gray matter changes can present a relatively scattered and heterogeneous spatial distribution [12,42]. H-MSResNet can simultaneously capture the local details and contextual information of a large spatial range by performing convolution operations with different receptive fields in parallel [43], thereby showing an enhanced ability to represent image features at different spatial scales. Liu et al. [44] applied parallel training of fixed-scale branches and variable-scale branches to process scale changes in images. Jeong et al. [32] also used parallel branches with two different sizes of convolution kernels, 1 × 3 and 1 × 7, to identify epileptogenic focus nodes in drug-resistant epilepsy in children, providing a methodological basis for its application in epilepsy image analysis.

Multiple metrics, including accuracy, sensitivity, specificity, F1 score, and AUC, should be considered jointly when evaluating medical imaging AI models, as a single metric is insufficient to characterize overall model performance [45]. In this study, H-MSResNet achieved higher mean accuracy, specificity, and F1 score than ResNet-34 and DenseNet-121, whereas its sensitivity and AUC were lower than those of both comparison models. At the evaluated decision threshold, the higher specificity together with the lower sensitivity reflects a different error profile: fewer healthy controls were misclassified as patients, whereas more patients were misclassified as healthy controls. In the present study, the higher specificity partly offset the lower sensitivity, resulting in a higher mean accuracy. In contrast, AUC reflects overall discrimination across a range of classification thresholds rather than performance at a single operating point. The lower AUC of H-MSResNet therefore indicates weaker overall discriminative ability across thresholds than ResNet-34 and DenseNet-121. The specific factors underlying this performance profile cannot be determined from the present experiments and require further investigation. From a clinical perspective, this profile suggests that H-MSResNet is more effective at reducing false-positive classifications than at minimizing missed patients at the evaluated decision threshold. This characteristic may be less favorable for diagnostic screening, where sensitivity and the avoidance of false-negative predictions are particularly important. These findings suggest that future model optimization should place greater emphasis on improving sensitivity and overall discrimination, while carefully evaluating the associated changes in specificity.

### 4.2. Clinical and Pathological Basis of Brain Regions Revealed by LRP

By quantitatively mapping the population-level LRP relevance maps to the Harvard–Oxford Atlas, we observed that model attribution was predominantly distributed across regions in the temporal lobe and limbic system. These attribution patterns reflect the anatomical distribution of image information contributing to the trained classifier’s decisions and should be interpreted as model-dependent explanations rather than direct evidence of radiologically visible lesions or pathological abnormalities.

Among the top 10 most important brain regions, 9 are located in the temporal lobe and its medial region. R_mTGad ranks first, followed by L_Amy. The anterior and posterior divisions of the parahippocampal gyrus are in the top 10 (5th on the left posterior side, 9th on the left anterior side). These regions are all classic anatomical structures associated with TLE. Previous studies showed that although these MRI-negative temporal lobe epilepsy patients lack structural lesions in routine visual examination, they may still have subtle morphological and microstructural abnormalities in the medial and lateral temporal lobe structures [46,47]. Further studies comparing volume measurements with histopathology confirmed that changes in the volume of the amygdala and hippocampus are also significantly correlated with postoperative pathological diagnosis [12,48]. In addition, the large-scale ENIGMA study further confirmed that structural changes in patients with TLE are mainly concentrated in the ipsilateral temporal lobe limbic system region [49]. Overall, the regional distribution identified by LRP broadly corresponded to the temporal and limbic regions implicated in these structural imaging studies. Recent explainable deep-learning studies provide additional context for these findings. Gleichgerrcht et al. reported that CNN saliency in MRI-negative TLE was prominently distributed in medial temporal regions, including the amygdala, hippocampus, and parahippocampus [17]. More recently, Kaestner et al. reported that saliency for TLE-versus-HC classification followed a limbic pattern involving the hippocampus, parahippocampal cortical regions, cingulate cortex, and lateral temporal regions in a large multicenter T1-weighted MRI study of TLE [50]. The temporal and limbic attribution patterns observed in the present LRP analysis are generally consistent with those reported in these previous explainable deep-learning studies, although direct comparison is limited by differences in study populations and attribution methods.

Outside the temporal lobe, the left frontal operculum cortex also ranked among the top 10 in the LRP importance ranking. The frontal operculum cortex is anatomically connected to the anterior temporal lobe structures via the uncinate process [51], suggesting a potential anatomical association with temporal lobe-related structures. Previous magnetoencephalography studies found that patients with medial TLE have significant abnormalities in the functional connectivity of the temporal and frontal lobe networks, and this abnormality is negatively correlated with the patients’ attention and working memory scores [52]. The findings on the left frontal operculum cortex suggest that the model may rely on the temporal lobe region and related structural information outside the temporal lobe during classification. However, this result only reflects the distribution of the model’s dependence on imaging features and cannot infer specific brain region interactions or pathological transmission mechanisms. Therefore, this region should be further validated as a potential candidate brain region for MRI-negative TLE.

This study further observed that the contribution distribution of different candidate brain regions was not entirely consistent between the left and right hemispheres, as shown in Figure 8. LRP showed a certain lateral distribution characteristic throughout the brain, with some brain regions exhibiting a bilaterally symmetrical high contribution pattern.

In the left-dominant pattern, the structures related to the medial limbic system showed relatively higher left-sided attribution scores. L_Amy is the furthest from the diagonal, with its left–right score difference ranking among the highest in the whole brain. Structures such as the anterior and posterior divisions of the left parahippocampal gyrus showed relatively higher left-sided attribution scores. This finding is consistent with the results of the ENIGMA-Epilepsy working group and Park et al. [8,53], who stated that patients with left-sided TLE show great damage to the medial temporal lobe structures and hemispheric asymmetry changes. By analyzing whole-brain gene expression data, Larivière et al. [54] further found that the structural changes related to focal epilepsy may have spatial consistency with the distribution of epilepsy risk gene expression, and this association is particularly significant in the left hemisphere. The results of the current work suggest that the model may have captured the imaging features related to this structural asymmetry.

In the right-dominant pattern, the lateral temporal lobe neocortex was highly prominent, with R_mTGad being the most important in the whole brain. The posterior division of the right superior temporal gyrus and R_iTGad also showed high weights. These regions exhibited consistent right-dominance in multiple individuals, suggesting that they may have a stable discriminative contribution in the classification of MRI-negative TLE. Intracranial EEG studies by Matoušková et al. [55] confirmed that the lateral temporal lobe cortex (including the middle temporal gyrus, superior temporal gyrus, and inferior temporal gyrus) plays a connecting role as a core network hub in the cognitive network. Structural network studies and resting-state functional magnetic resonance imaging studies further found that the node efficiency and topology of the lateral temporal lobe region in TLE patients are impaired as a whole, the functional synchronicity between hemispheres is widely reduced, and the structural and functional topology of this region may be altered [56,57]. The results of the current work are consistent with those of the above multimodal studies in terms of spatial distribution. This finding indicates that the model may rely on structural imaging features involving the right lateral temporal regions during classification.

In the bilateral balanced model, the brain regions represented by the posterior division of the inferior temporal gyrus showed high and similar contribution values in both hemispheres, exhibiting a near diagonal distribution in Figure 8. Although these regions showed relatively balanced attribution scores between the two hemispheres, their absolute bilateral contribution was at a high level in the whole brain, suggesting that they may have stable bilateral information expression characteristics in model classification. This result indicates that the classification-related imaging information of MRI-negative TLE is not entirely limited to unilateral structures but may involve a certain degree of bilateral co-involvement.

In summary, the population analysis results based on LRP indicate that H-MSResNet primarily relies on temporal lobe and limbic system structures and involves a small number of extratemporal regions in the classification of MRI-negative TLE. Furthermore, the distribution of these contributing regions exhibits interhemispheric differences and partially bilateral symmetric characteristics. These results suggest that subtle discriminative information distributed across multiple anatomical regions may exist in the structural images of MRI-negative TLE, providing candidate brain region clues for subsequent morphological analysis and multimodal imaging studies.

### 4.3. Limitations and Future Directions

This study still has certain limitations. First, all participants were recruited from a single center, and no independent multicenter dataset or data acquired using different scanners or acquisition protocols were available for external validation. Therefore, the reported performance should be interpreted as internal validation and does not establish generalizability across institutions or heterogeneous scanning conditions. Further validation in independent multicenter cohorts is required. Second, the present computational analysis was based solely on T1-weighted MRI and did not incorporate other structural MRI sequences, such as FLAIR or T2-weighted imaging, or complementary modalities such as diffusion or functional MRI. Future studies integrating multisequence or multimodal MRI may provide a more comprehensive assessment of MRI-negative TLE.

Third, LRP-based attribution depends on the propagation rule and stabilization parameter. Although the ε-rule (ε = 1 × 10^−8^) was used to improve numerical stability, the resulting relevance values may still be affected by baseline shifts or other numerical effects. Therefore, the regional attribution results should be interpreted as method-dependent, and further robustness evaluation across different LRP configurations is warranted. Fourth, complete seizure-onset laterality information was not available for the entire cohort, precluding reliable stratified classification and LRP attribution analyses for left- and right-sided TLE. Therefore, the observed interhemispheric attribution differences should not be interpreted as direct evidence of seizure-focus lateralization. In addition, surgical outcomes and histopathological data were not systematically available for the entire cohort, precluding further clinicopathological validation of the imaging findings. Future studies with more complete clinical characterization are needed to evaluate the relationships between model attribution patterns, seizure-onset laterality, and clinical or pathological outcomes.

Fifth, resampling the Harvard–Oxford Atlas may introduce spatial correspondence errors, particularly at regional boundaries, and its limited spatial resolution may obscure fine-grained regional differences. Future studies may further evaluate the robustness of regional attribution using alternative atlases or higher-resolution anatomical parcellations.

## 5. Conclusions

This study constructed a framework based on a hierarchical, multiscale 3D residual network and LRP for identifying MRI-negative temporal lobe epilepsy. The model achieved an average classification accuracy of 76.27%, validating the ability of multiscale feature extraction strategies to extract subtle structural imaging information associated with MRI-negative TLE. LRP revealed key brain regions highly concentrated in the parahippocampal gyrus, amygdala, and multiple temporal lobe subregions, with relatively high attribution from the left frontal operculum cortex and other areas. Whole-brain analysis showed that the brain regions relied upon by the model exhibited a distribution pattern of lateralization and bilateral symmetry. Some medial limbic system structures were left-dominant, some lateral temporal lobe neocortical structures were right-dominant, and symmetrical brain regions with high bilateral importance scores were also present. This study preliminarily validated that the combination of deep learning methods and LRP interpretability analysis can achieve an accurate classification of MRI-negative epilepsy while revealing the key brain regions the model relies on for its decisions.

## Figures and Tables

**Table 1:** Demographic and clinical characteristics of the study participants.

Characteristic	MRI-Negative TLE (*n* = 101)	Healthy Controls (*n* = 101)	*p* Value
Age, years	33.58 ± 13.34	37.46 ± 15.19	0.056
Sex, male/female	57/44	53/48	0.572
Disease duration, years	11.71 ± 9.74	—	—

**Table 2:** Performance comparison of three 3D-CNN models using fivefold cross-validation.

Model	Average Accuracy	Best Accuracy	Average Sensitivity	Average Specificity	Average F1 Score	Average AUC
ResNet-34	0.7426 ± 0.0120	0.7561	0.7705 ± 0.1509	0.7129 ± 0.1624	0.7435 ± 0.0422	0.7766 ± 0.0353
DenseNet-121	0.7474 ± 0.0433	0.8000	0.7533 ± 0.0859	0.7419 ± 0.1328	0.7485 ± 0.0361	0.7820 ± 0.0659
H-MSResNet	0.7627 ± 0.0443	0.8250	0.7333 ± 0.0901	0.7919 ± 0.0588	0.7531 ± 0.0564	0.7415 ± 0.0417

## Data Availability

The datasets used and analysed during the current study are available from the corresponding authors on reasonable request.

## Appendix A

**Table A1:** Comparison of English abbreviations and full names for the Harvard–Oxford Cortical and Subcortical Atlases.

Region	Abbreviation	Full Name	Region	Abbreviation	Full Name
Cortical	FP	Frontal Pole	Cortical	CGad	Cingulate Gyrus, anterior division
Cortical	InsC	Insular Cortex	Cortical	CGpd	Cingulate Gyrus, posterior division
Cortical	sFG	Superior Frontal Gyrus	Cortical	PC	Precuneous Cortex
Cortical	mFG	Middle Frontal Gyrus	Cortical	CC	Cuneal Cortex
Cortical	iFGptri	Inferior Frontal Gyrus, pars triangularis	Cortical	FOC	Frontal Orbital Cortex
Cortical	iFGpoper	Inferior Frontal Gyrus, pars opercularis	Cortical	PGad	Parahippocampal Gyrus, anterior division
Cortical	PrecG	Precentral Gyrus	Cortical	PGpd	Parahippocampal Gyrus, posterior division
Cortical	TP	Temporal Pole	Cortical	LG	Lingual Gyrus
Cortical	sTGad	Superior Temporal Gyrus, anterior division	Cortical	TFCad	Temporal Fusiform Cortex, anterior division
Cortical	sTGpd	Superior Temporal Gyrus, posterior division	Cortical	TFCpd	Temporal Fusiform Cortex, posterior division
Cortical	mTGad	Middle Temporal Gyrus, anterior division	Cortical	TOFC	Temporal Occipital Fusiform Cortex
Cortical	mTGpd	Middle Temporal Gyrus, posterior division	Cortical	OFG	Occipital Fusiform Gyrus
Cortical	mTGTP	Middle Temporal Gyrus, temporooccipital part	Cortical	FopC	Frontal Operculum Cortex
Cortical	iTGad	Inferior Temporal Gyrus, anterior division	Cortical	copC	Central Opercular Cortex
Cortical	iTGpd	Inferior Temporal Gyrus, posterior division	Cortical	PopC	Parietal Operculum Cortex
Cortical	iTGTP	Inferior Temporal Gyrus, temporooccipital part	Cortical	PP	Planum Polare
Cortical	PostcG	Postcentral Gyrus	Cortical	HG	Heschl’s Gyrus (includes H1 and H2)
Cortical	sPL	Superior Parietal Lobule	Cortical	PT	Planum Temporale
Cortical	SGad	Supramarginal Gyrus, anterior division	Cortical	SpcC	Supracalcarine Cortex
Cortical	SGpd	Supramarginal Gyrus, posterior division	Cortical	OP	Occipital Pole
Cortical	AG	Angular Gyrus	Subcortical	T	Thalamus
Cortical	LOCsd	Lateral Occipital Cortex, superior division	Subcortical	C	Caudate
Cortical	LOCid	Lateral Occipital Cortex, inferior division	Subcortical	Put	Putamen
Cortical	IcalC	Intracalcarine Cortex	Subcortical	Pal	Pallidum
Cortical	FMC	Frontal Medial Cortex	Subcortical	B	Brain-Stem
Cortical	JLC	Juxtapositional Lobule Cortex (formerly Supplementary Motor Cortex)	Subcortical	H	Hippocampus

## References

[1] Fisher R.S., van Emde Boas W., Blume W., Elger C., Genton P., Lee P., Engel J. (2005). Epileptic seizures and epilepsy: Definitions proposed by the International League Against Epilepsy (ILAE) and the International Bureau for Epilepsy (IBE). Epilepsia.

[2] Thijs R.D., Surges R., O’Brien T.J., Sander J.W. (2019). Epilepsy in adults. Lancet.

[3] Duncan J.S., Winston G.P., Koepp M.J., Ourselin S. (2016). Brain imaging in the assessment for epilepsy surgery. Lancet Neurol..

[4] Jones A.L., Cascino G.D. (2016). Evidence on use of neuroimaging for surgical treatment of temporal lobe epilepsy: A systematic review. JAMA Neurol..

[5] Muhlhofer W., Tan Y.L., Mueller S.G., Knowlton R. (2017). MRI-negative temporal lobe epilepsy-What do we know?. Epilepsia.

[6] Bernasconi A., Bernasconi N., Bernhardt B.C., Schrader D. (2011). Advances in MRI for ‘cryptogenic’ epilepsies. Nat. Rev. Neurol..

[7] Bien C.G., Szinay M., Wagner J., Clusmann H., Becker A.J., Urbach H. (2009). Characteristics and surgical outcomes of patients with refractory magnetic resonance imaging-negative epilepsies. Arch. Neurol..

[8] Whelan C.D., Altmann A., Botía J.A., Jahanshad N., Hibar D.P., Absil J., Alhusaini S., Alvim M.K.M., Auvinen P., Bartolini E. (2018). Structural brain abnormalities in the common epilepsies assessed in a worldwide ENIGMA study. Brain.

[9] Yang L., Peng B., Gao W., A R., Liu Y., Liang J., Zhu M., Hu H., Lu Z., Pang C. (2024). Automated detection of MRI-negative temporal lobe epilepsy with ROI-based morphometric features and machine learning. Front. Neurol..

[10] Yang W., Niu J., Xiong Y., Wang X., Li J., Wang Z., Song D., Chen B. (2025). Gray matter microstructural abnormalities in temporal lobe epilepsy with hippocampal sclerosis and MRI negative. Eur. J. Radiol..

[11] Blackmon K., Barr W.B., Morrison C., MacAllister W., Kruse M., Pressl C., Wang X., Dugan P., Liu A.A., Halgren E. (2019). Cortical gray-white matter blurring and declarative memory impairment in MRI-negative temporal lobe epilepsy. Epilepsy Behav..

[12] Ballerini A., Casarini A., Biagioli N., Mirandola L., Ballotta D., Summers P., Scolastico S., Madrassi L., Genovese M., Malagoli M. (2026). Multidimensional profiling of MRI-negative temporal lobe epilepsy uncovers distinct phenotypes. Ann. Clin. Transl. Neurol..

[13] Wen J., Thibeau-Sutre E., Diaz-Melo M., Samper-González J., Routier A., Bottani S., Dormont D., Durrleman S., Burgos N., Colliot O. (2020). Convolutional neural networks for classification of Alzheimer’s disease: Overview and reproducible evaluation. Med. Image Anal..

[14] Pan D., Zeng A., Yang B., Lai G., Hu B., Song X., Jiang T. (2023). Deep learning for brain MRI confirms patterned pathological progression in Alzheimer’s disease. Adv. Sci..

[15] Chang A.J., Roth R., Bougioukli E., Ruber T., Keller S.S., Drane D.L., Gross R.E., Welsh J., Abrol A., Calhoun V. (2023). MRI-based deep learning can discriminate between temporal lobe epilepsy, Alzheimer’s disease, and healthy controls. Commun. Med..

[16] Kaestner E., Hassanzadeh R., Gleichgerrcht E., Hasenstab K., Roth R.W., Chang A., Rüber T., Davis K.A., Dugan P., Kuzniecky R. (2024). Adding the third dimension: 3D convolutional neural network diagnosis of temporal lobe epilepsy. Brain Commun..

[17] Gleichgerrcht E., Kaestner E., Hassanzadeh R., Roth R.W., Parashos A., Davis K.A., Bagić A., Keller S.S., Rüber T., Stoub T. (2025). Redefining diagnostic lesional status in temporal lobe epilepsy with artificial intelligence. Brain.

[18] Houssein E.H., Gamal A.M., Younis E.M., Mohamed E. (2025). Explainable artificial intelligence for medical imaging systems using deep learning: A comprehensive review. Clust. Comput..

[19] Mir A.N., Rizvi D.R. (2025). Advancements in deep learning and explainable artificial intelligence for enhanced medical image analysis: A comprehensive survey and future directions. Eng. Appl. Artif. Intell..

[20] Mehmood A., Mehmood F., Kim J. (2025). Towards Explainable Deep Learning in Computational Neuroscience: Visual and Clinical Applications. Mathematics.

[21] Borys K., Schmitt Y.A., Nauta M., Seifert C., Krämer N., Friedrich C.M., Nensa F. (2023). Explainable AI in medical imaging: An overview for clinical practitioners—Beyond saliency-based XAI approaches. Eur. J. Radiol..

[22] Saw S.N., Yan Y.Y., Ng K.H. (2025). Current status and future directions of explainable artificial intelligence in medical imaging. Eur. J. Radiol..

[23] Jin W., Li X., Fatehi M., Hamarneh G. (2023). Guidelines and evaluation of clinical explainable AI in medical image analysis. Med. Image Anal..

[24] Kim B., Seo J., Jeon S., Koo J., Choe J., Jeon T. Why are saliency maps noisy? cause of and solution to noisy saliency maps. Proceedings of the 2019 IEEE/CVF International Conference on Computer Vision Workshop (ICCVW).

[25] Bach S., Binder A., Montavon G., Klauschen F., Müller K.-R., Samek W. (2015). On pixel-wise explanations for non-linear classifier decisions by layer-wise relevance propagation. PLoS ONE.

[26] Böhle M., Eitel F., Weygandt M., Ritter K. (2019). Layer-wise relevance propagation for explaining deep neural network decisions in MRI-based Alzheimer’s disease classification. Front. Aging Neurosci..

[27] Eitel F., Soehler E., Bellmann-Strobl J., Brandt A.U., Ruprecht K., Giess R.M., Kuchling J., Asseyer S., Weygandt M., Haynes J.-D. (2019). Uncovering convolutional neural network decisions for diagnosing multiple sclerosis on conventional MRI using layer-wise relevance propagation. NeuroImage Clin..

[28] Wang D., Honnorat N., Fox P.T., Ritter K., Eickhoff S.B., Seshadri S., Habes M. (2023). Deep neural network heatmaps capture Alzheimer’s disease patterns reported in a large meta-analysis of neuroimaging studies. NeuroImage.

[29] Kim D., Lee J., Moon J., Moon T. (2022). Interpretable deep learning-based hippocampal sclerosis classification. Epilepsia Open.

[30] Gao S.H., Cheng M.M., Zhao K., Zhang X.Y., Yang M.H., Torr P. (2021). Res2Net: A new multi-scale backbone architecture. IEEE Trans. Pattern Anal. Mach. Intell..

[31] Harouni M., Goyal V., Feldman G., Michael S., Voss T.C. (2025). Deep multi-scale and attention-based architectures for semantic segmentation in biomedical imaging. Comput. Mater. Contin..

[32] Jeong J.W., Lee M.H., Kuroda N., Sakakura K., O’Hara N., Juhasz C., Asano E. (2022). Multi-scale deep learning of clinically acquired multi-modal MRI improves the localization of seizure onset zone in children with drug-resistant epilepsy. IEEE J. Biomed. Health Inform..

[33] Dale A.M., Fischl B., Sereno M.I. (1999). Cortical surface-based analysis. I. segmentation and surface reconstruction. NeuroImage.

[34] Fischl B., Sereno M.I., Dale A.M. (1999). Cortical surface-based analysis. II: Inflation, flattening, and a surface-based coordinate system. NeuroImage.

[35] Fischl B., Salat D.H., Busa E., Albert M., Dieterich M., Haselgrove C., van der Kouwe A., Killiany R., Kennedy D., Klaveness S. (2002). Whole brain segmentation: Automated labeling of neuroanatomical structures in the human brain. Neuron.

[36] He K., Zhang X., Ren S., Sun J. Deep residual learning for image recognition. Proceedings of the 2016 IEEE Conference on Computer Vision and Pattern Recognition (CVPR).

[37] Xu W., Fu Y.L., Zhu D. (2023). ResNet and its application to medical image processing: Research progress and challenges. Comput. Methods Programs Biomed..

[38] Huang G., Liu Z., Van Der Maaten L., Weinberger K.Q. Densely connected convolutional networks. Proceedings of the 2017 IEEE Conference on Computer Vision and Pattern Recognition (CVPR).

[39] Kohlbrenner M., Bauer A., Nakajima S., Binder A., Samek W., Lapuschkin S. Towards best practice in explaining neural network decisions with LRP. Proceedings of the 2020 International Joint Conference on Neural Networks (IJCNN).

[40] Shojaei S., Saniee Abadeh M., Momeni Z. (2023). An evolutionary explainable deep learning approach for Alzheimer’s MRI classification. Expert Syst. Appl..

[41] Nilearn Contributors (2025). nilearn.

[42] Riederer F., Lanzenberger R., Kaya M., Prayer D., Serles W., Baumgartner C. (2008). Network atrophy in temporal lobe epilepsy: A voxel-based morphometry study. Neurology.

[43] Yu F., Koltun V. Multi-scale context aggregation by dilated convolutions. Proceedings of the International Conference on Learning Representations (ICLR).

[44] Liu Y., Zhong Y., Qin Q. (2018). Scene classification based on multiscale convolutional neural network. IEEE Trans. Geosci. Remote Sens..

[45] Klontzas M.E., Groot Lipman K.B.W., Akinci D’Antonoli T., Andreychenko A., Cuocolo R., Dietzel M., Gitto S., Huisman H., Santinha J., Vernuccio F. (2026). ESR essentials: Common performance metrics in AI—Practice recommendations by the European Society of Medical Imaging Informatics. Eur. Radiol..

[46] Vaughan D.N., Rayner G., Tailby C., Jackson G.D. (2016). MRI-negative temporal lobe epilepsy: A network disorder of neocortical connectivity. Neurology.

[47] Bernhardt B.C., Worsley K.J., Besson P., Concha L., Lerch J.P., Evans A.C., Bernasconi N. (2008). Mapping limbic network organization in temporal lobe epilepsy using morphometric correlations: Insights on the relation between mesiotemporal connectivity and cortical atrophy. NeuroImage.

[48] Heers M., Schumacher K.F., Doostkam S., Rohrer Y., Yildirim C., Altenmüller D.-M., Staack A.M., Scheiwe C.F., Reinacher P.C., Steinhoff B.J. (2025). Association of amygdala and hippocampus volumes with histopathological diagnosis in patients with temporal lobe epilepsy. Neurol. Open Access.

[49] Larivière S., Rodríguez-Cruces R., Royer J., Caligiuri M.E., Gambardella A., Concha L., Keller S.S., Cendes F., Yasuda C., Bonilha L. (2020). Network-based atrophy modeling in the common epilepsies: A worldwide ENIGMA study. Sci. Adv..

[50] Kaestner E., Sawant J., Arienzo D., Hasenstab K.A., Gleichgerrcht E., Gholipour T., Abrol A., Hassanzadeh R., Thomopoulos S.I., Yasuda C.L. (2026). Disease detection and classification in temporal lobe epilepsy: Step-wise versus simultaneous AI decision models in a multisite neuroimaging study. Brain Commun..

[51] Von Der Heide R.J., Skipper L.M., Klobusicky E., Olson I.R. (2013). Dissecting the uncinate fasciculus: Disorders, controversies and a hypothesis. Brain.

[52] Ishizaki T., Maesawa S., Suzuki T., Hashida M., Ito Y., Yamamoto H., Tanei T., Natsume J., Hoshiyama M., Saito R. (2025). Frequency-specific network changes in mesial temporal lobe epilepsy: Analysis of chronic and transient dysfunctions in the temporo-amygdala-orbitofrontal network using magnetoencephalography. Epilepsia Open.

[53] Park B.-Y., Larivière S., Rodríguez-Cruces R., Royer J., Tavakol S., Wang Y., Caciagli L., Caligiuri M.E., Gambardella A., Concha L. (2022). Topographic divergence of atypical cortical asymmetry and atrophy patterns in temporal lobe epilepsy. Brain.

[54] Larivière S., Royer J., Rodríguez-Cruces R., Paquola C., Caligiuri M.E., Gambardella A., Concha L., Keller S.S., Cendes F., Yasuda C.L. (2022). Structural network alterations in focal and generalized epilepsy assessed in a worldwide ENIGMA study follow axes of epilepsy risk gene expression. Nat. Commun..

[55] Matouskova B., Cimbalnik J., Daniel P., Kojan M., Roman R., Jurkovicova L., Pail M., Brazdil M., Kucewicz M., Klimes P. (2026). Network hubs supporting memory encoding: Implications for cognitive preservation in epilepsy surgery. Epilepsia.

[56] Ge Y., Chen C., Li H., Wang R., Yang Y., Ye L., He C., Chen R., Wang Z., Shao X. (2024). Altered structural network in temporal lobe epilepsy with focal to bilateral tonic–clonic seizures. Ann. Clin. Transl. Neurol..

[57] Wu J., Wu J., Guo R., Chu L., Li J., Zhang S., Ren H. (2022). The decreased connectivity in middle temporal gyrus can be used as a potential neuroimaging biomarker for left temporal lobe epilepsy. Front. Psychiatry.

